# Enhancing the Evaluation of Physical Function Following Orthopaedic Trauma Care: Comparison of PROMIS Computerized Adaptive Testing and Short Musculoskeletal Function Assessment

**DOI:** 10.1097/BOT.0000000000002814

**Published:** 2024-05-31

**Authors:** Michiel A. J. Luijten, Lotte Haverman, Caroline B. Terwee, Martijn Poeze, Diederik O. Verbeek

**Affiliations:** a Department of Epidemiology and Data Science, Amsterdam UMC Location Vrije Universiteit, Amsterdam, The Netherlands;; b Department of Child and Adolescent Psychiatry and Psychosocial Care, Emma Children's Hospital, Amsterdam UMC Location AMC, Amsterdam, The Netherlands;; c Amsterdam Public Health Research Institute, Methodology, Amsterdam, The Netherlands;; d Amsterdam Public Health Research Institute, Mental Health and Digital Health Amsterdam, The Netherlands; and; e Department of Surgery, Division of Trauma, Maastricht University Medical Center+, Maastricht, The Netherlands.

**Keywords:** PROMIS, CAT, patient-reported outcomes, lower extremity, upper extremity, physical function

## Abstract

**OBJECTIVES:**

To compare measurement properties of Patient-Reported Outcomes Measurement Information System (PROMIS) (physical function [PF] and pain interference [PI]) computerized adaptive testing to traditional Short Musculoskeletal Function Assessment (SMFA) (dysfunction index [DI] and bother index [BI]). To explore factors associated with PROMIS scores.

**METHODS:**

**Design:**

Cross-sectional study.

**Setting:**

Level I Trauma Center.

**Patient Selection Criteria:**

Isolated upper/lower extremity fracture patients were recruited from the orthopaedic trauma outpatient clinic (October 1, 2021 to January 1, 2023).

**Outcome Measures:**

Correlations (Pearson), reliability (standard error [SE] [T score]), efficiency (amount of information per item [1 − SE^2^/N_items_]), and floor/ceiling effects were assessed. An r > 0.7 represented high correlation, and SE ≤ 2.2 represented sufficient reliability. Factors associated with worse PROMIS scores were also identified.

**RESULTS:**

In total, 202 patients completed PROMs at median 98 days follow-up. Correlations between PROMIS-PF and SMFA-DI, and PROMIS-PI and SMFA-BI were −0.84 and 0.65. Reliability was very high for both instruments (mean SE 2.0 [PROMIS-PF], SE 2.1 [PROMIS-PI], and SE 1.2 [SMFA-DI], SE 1.8 [SMFA-BI]). Relative efficiency for PROMIS-PF versus SMFA-DI, and PROMIS-PI versus SMFA-BI was 7.8 (SD 2.5) and 4.1 (SD 1.7), respectively. Neither PROMIS nor SMFA exhibited floor/ceiling effects. In the multivariable regression analyses, elevated levels of depression, among other factors, showed an (independent) association with worse PROMIS-PF and PROMIS-PI scores.

**CONCLUSIONS:**

PROMIS-PF and PROMIS-PI CATs showed a (high and moderate) correlation with SMFA and hence measure a comparable construct of physical function and discomfort. As computerized adaptive tests are much more efficient to administer, they present a compelling alternative to SMFA for evaluating impact of fracture treatment. The relation between symptoms of depression and PROMIS scores emphasizes the importance of psychosocial aspects of health in orthopaedic trauma patients.

**LEVEL OF EVIDENCE:**

Prognostic Level III. See Instructions for Authors for a complete description of levels of evidence.

## INTRODUCTION

As health systems worldwide move toward patient-centered and outcome-based care, the need for efficient instruments to accurately measure outcomes from the patients' perspective is increasingly recognized. Along with clinical and radiographic outcomes, patient-reported outcome measures (PROMs) are now considered essential tools to evaluate success of orthopaedic interventions.^1,2^ Moreover, there is a mounting belief that broader implementation of PROMs in the clinical setting and in large registries is fundamental to further improve quality of care and advance the value of health care delivery.^3–7^

The Short Musculoskeletal Function Assessment (SMFA) is an American Academy of Orthopaedic Surgeons–endorsed PROM that is traditionally used to evaluate patients' overall physical function in orthopaedic trauma populations.^8,9^ Its common use notwithstanding, the SMFA has been found to have suboptimal performance in certain patient groups and takes considerable time to complete.^10–15^ This is important as “questionnaire fatigue” among patients is a common concern, particularly when PROMs use is considered in daily clinical practice.

A suitable universally applicable alternative to the SMFA may be the Patient-Reported Outcomes Measurement Information System (PROMIS).^16^ This National Institute of Health–funded measurement system was developed to efficiently assess and report common symptoms and physical, mental, and social functioning across patient populations. Since its launch in 2004, the use of PROMIS has slowly gained ground across the world. Outside the United States, the Netherlands has been at the forefront of PROMIS implementation and its questionnaires are available in Dutch–Flemish for over 10 years.^17–19^ PROMIS consists of item (question) banks assessing different domains of symptoms and functioning. Of those, Physical Function (PROMIS-PF) and Pain Interference (PROMIS-PI) are considered especially relevant for orthopaedic trauma patients.^6,20,21^

Previous studies in general orthopaedics have shown PROMIS measures to compare favorably to a number of traditionally used “legacy” PROMs, especially when administered as a computerized adaptive test (CAT).^22,23^ This flexible method, based on item response theory, allows for the most relevant questions to be selected electronically from PROMIS item banks. Overall, PROMIS CATs have been found to correlate well with a range of legacy instruments, while taking considerably less time to complete than fixed item (paper) questionnaires.^22,23^ Still, PROMIS CATs remain infrequently used in orthopaedic trauma patients, and their performance relative to the SMFA in this population has not been fully defined.^24^

Therefore, this study's primary objective was to correlate PROMIS-PF and PROMIS-PI CATs to the SMFA and compare reliability and floor and ceiling effects. We hypothesized that PROMIS (physical function [PF] and pain interference [PI]) CATs would be highly correlated with the SMFA but would be more efficient (in terms of completion time and information obtained per item) to administer. Furthermore, we hypothesized that PROMIS (PF and PI) CATs would not exhibit floor or ceiling effects, while a ceiling effect was expected for the SMFA.

Our secondary objective was to explore factors associated with PROMIS (PF and PI) CAT scores. For this purpose, we included sociodemographic and fracture specific factors as well as depressive symptoms and pain intensity.

## METHODS

### Design

Institutional review board approval was obtained for this prospective, cross-sectional, single center study. Patients were recruited from the orthopaedic trauma outpatient clinic of an Academic Level I trauma center in a 15-month period (October 1, 2021–January 1, 2023).

### Patients

Eligible for study inclusion were adult patients with at least 1 month follow-up for an operatively or nonoperatively treated acute and isolated fracture of the upper or lower extremity, and who had no weightbearing or motion restrictions. Excluded were patients younger than 18 years of age, multiply injured patients, and patients with cognitive impairments or language impediments, who were unable to understand or complete questionnaires in Dutch.

Eligible participants were identified from the daily clinic appointment list by one of the investigators (D.O.V.) and subsequently approached to explain study details. Patients who agreed to participate provided informed consent and were instructed on the use of the touchscreen computer tablet (Apple iPad tablet [Cupertino, CA]) with wireless connectivity.

In total, 642 patients were deemed eligible for study inclusion. Of those, 202 (31%) patients agreed to participate.

Patient characteristics and PROMs (PROMIS CATs and SMFA) were collected using an online (“KLIK”) PROM portal accessed on the computer tablet.^25^

### Patient Characteristics

Sociodemographic data were collected from all patients, and fracture specifics were identified from electronic health records. Patients' depressive symptoms were measured using the Dutch–Flemish PROMIS Depression CAT (version 1.0), consisting of a variable selection of items per patient from a total of 28 items.^26^ Patients were also asked to rate their pain intensity at the time of enrollment as no, slight, moderate, severe, or extreme pain.

### PROMIS CATs

The Dutch–Flemish PROMIS item banks for PF (version 1.2) and PI (version 1.1) consist of a total of 121 and 40 items, respectively.^17–19^ PROMIS item banks were administered as CATs. Each item is scored on a 5-point Likert scale. Physical function items assess mobility, upper extremity, and axial (neck and back) function, as well as activities of daily living, among others. Pain interference items evaluate to which extent and how often pain hinders engagement in certain functions such as recreational and social activities. CATs were programmed to stop when either a standard error (SE) of ≤2.2 (corresponding with a 95% reliability) was reached or when a maximum of 12 items was administered. CATs always administered a minimum of 2 items. PROMIS scores are expressed on a T score metric with a mean of 50 and an SD of 10 in the US population (the mean T score in the Dutch population is also 50).^27^ Higher scores represent more of the construct measured, in other words, better physical function for PF and increased pain interference for PI.

### Short Musculoskeletal Function Assessment

The SMFA assesses overall physical functioning, and contains a total of 46 items that are scored on a 5-point Likert scale.^9^ It consists of 2 indices, the dysfunction index (DI) (34 items), and the bother index (BI) (12 items). The DI evaluates a patient's perceived performance when engaging in certain functions and (daily) activities. Both the amount of difficulty patients experience and how often patients have difficulty are assessed. The BI provides insight into how much patients are bothered by problems in an array of functional areas (such as work and recreation). SMFA scores are calculated by summing the responses to the individual items and transforming these such that the range of scores is from 0 to 100, with higher scores indicating poorer function.

To allow comparison of efficiency between both PROMs, time to completion was electronically logged in seconds for PROMIS (PF and PI) CATs and the complete SMFA. Also, the mean number of PROMIS CAT items needed for test completion (N_items_) was recorded for each patient. PROMs were completed in variable order to correct for response fatigue.

### Statistics

#### Correlation

Pearson correlations between PROMIS (PF and PI) CATs and SMFA (DI and BI) were calculated. A high correlation (r > 0.7) was expected between PROMIS-PF CAT and SMFA-DI, as both instruments specifically assess overall physical functioning.^28^ A high correlation (r > 0.7) was also expected between PROMIS-PI CAT and SMFA-BI, as both instruments assess discomfort, although PI is more focused specifically on the element of pain.

#### Reliability

Reliability was based on a single measurement and therefore refers to internal reliability or internal consistency. To compare instruments, we calculated thetas and accompanying standard errors (SE) for each individual by fitting an item response theory model to the data. We used a graded response model to estimate item response theory parameters for the SMFA (DI and BI), while keeping the PROMIS (PF and PI) parameters fixed (also known as fixed parameter calibration). This ensures that both instruments are on the same scale and thus comparable. The SEs were calculated using the Expected A Posteriori estimator for all individuals for both PROMIS (PF and PI) and the SMFA (DI and BI). Next, we evaluated the mean SEs for the total sample. An SE of 2.2 corresponds with a 95% reliability and was considered sufficient. For the SMFA-DI and BI, we also calculated the Cronbach's alpha. An alpha between 0.70 and 0.95 was considered sufficient internal consistency.^29^

#### Efficiency

Efficiency of PROMs (or the amount of information obtained from each item) was calculated according to a division formula using the SE and number of items administered for each patient (1 − SE^2^/N_items_), and subsequently taking the mean of all patients.^30^ Relative efficiency was used to compare efficiency between the PROMIS CATs and the SMFA. For this purpose, we divided the mean efficiency of PROMIS (PF and PI) CATs by their associated counterpart of the SMFA (DI and BI).

#### Floor and Ceiling Effects

Test scores were also evaluated for floor (scores reflecting the lowest level of health possible) and ceiling (scores reflecting highest level of health possible) effects. An instrument was considered to have floor or ceiling effects if more than 15% of the participants selected the lowest or highest response on all administered items.^29,31^

#### Associated Factors

Univariable and multivariable linear regression analyses were performed to identify factors with the strongest association with worse PROMIS (PF and PI) CAT scores (corresponding with lower PF and higher PI scores). Variables included in the analysis were all sociodemographic and fracture specific factors as well as depressive symptoms and pain intensity. Variables with *P* < 0.10 in the univariable analyses were subsequently included in the multivariable linear regression analysis. Variables that did not meet this criterion and were excluded from the final model were country of birth and social status for both PROMIS-PF and PROMIS-PI CATs, and, additionally, gender, education level, employment, and treatment for PROMIS-PI CATs. Variables were evaluated for multicollinearity. None of the variables showed a high correlation (0.8 or greater). We also inspected assumptions of normality and linearity through histogram, probability–probability (P–P) plots, residual plots, and scatter plots, and all assumptions for performing linear regression analysis were met.

Standardized regression coefficients (β) were used to report results, which indicate the relative strength of the association between the various independent variables and patient reported outcome measure scores. The greater the absolute value of the β coefficient, the stronger the association.

The adjusted R-squared was calculated to measure the collective association of variables in the multivariate regression model with the PROMIS (PF and PI) CAT scores.

Data were analyzed using the Statistical Package for the Social Sciences (SPSS) version 25 (SPSS, Chicago, IL). For the item response theory analyses, we used R v2.4.1. and the R-package “mirt.”

## RESULTS

### Study Population

Mean age of the study cohort of 202 patients was 53 (SD 20; range 18–91), the majority was female (55%), had sustained a fracture of the foot/ankle or hand/wrist (54%), and received operative fracture fixation (80%) (Tables 1 and 2).

**TABLE 1.** Sociodemographic Variables (n = 202) (%)

Mean age, y (SD; range)	53 (20; 18–91)
Sex	
Male	92 (45)
Female	110 (55)
Country of birth	
Netherlands	193 (95)
Other	9 (5)
Education level	
Low	40 (20)
Intermediate	92 (45)
High	70 (35)
Employment status	
Paid employment	89 (45)
Unemployed	7 (4)
Worker compensation	27 (13)
Unpaid work	20 (10)
Studying	12 (6)
Retired	47 (23)
Social status	
Married/living with partner	128 (63)
Single/separated/widow	50 (25)
Living with parents	24 (12)

**TABLE 2.** Fracture Specifics (n = 202) (%)

Upper extremity	
Hand/wrist	41 (21)
Shoulder	17 (8)
Other arm	29 (14)
Lower extremity	
Foot/ankle	66 (33)
Hip	19 (9)
Other leg	30 (15)
Treatment	
Operative	162 (80)
Nonoperative	40 (20)
Median follow-up, d (range)	98 (36–4691)

### Correlation

The correlation between PROMIS-PF and SMFA-DI was −0.84, and the correlation between PROMIS-PI and SMFA-BI was 0.65 (Table 3). This was largely in accordance with the a priori defined hypothesis. The correlation between PROMIS-PI and the SMFA-BI was however slightly lower than expected.

**TABLE 3.** Pearson Coefficients (Mean N_items_)

PRO Measures	SMFA (46)	
PROMIS	Dysfunction index (34)	Bother index (12)
Physical function (5)	−0.84	−0.77
Pain interference (4)	0.61	0.65

### Reliability

The reliability for both PROMs was very high, as indicated by a mean SE of 2.0 and 2.1 for PROMIS-PF and PROMIS-PI CATs and a mean SE of 1.2 and 1.8 for SMFA-DI and BI, respectively. The Cronbach's alpha for the SMFA-DI and BI was 0.96 and 0.93, respectively.

### Efficiency

The mean number of items needed for PROMIS-PF and PROMIS-PI CATs was 5 (SD 2; range 3–12) and 4 (SD 3; range 2–12), respectively, compared with a fixed total of 46 items for the SMFA (DI 34, BI 12).

Relative efficiency for the PROMIS-PF CAT compared with the SMFA-DI was 7.8 (SD 2.5; range 2.5–11.2) and 4.1 (SD 1.7; range 0.8–9.2) for the PROMIS-PI CAT compared with the SMFA-BI. This indicates that the administered PROMIS CAT items provided, on average, 7.8 and 4.1 times more information per item compared with the SMFA.

Time to complete was mean 83 (SD 71) seconds for the PROMIS-PF CAT and 58 (SD 78) seconds for the PROMIS-PI CAT. Both CATs combined took mean 142 (SD 114) seconds. Completion time was much shorter for the PROMIS CATs compared with the SMFA (mean 357, median 343 (range 72–849) seconds).

### Floor and Ceiling Effects

Neither PROMIS (PF and PI) CATs nor the SMFA (DI and BI) exhibited floor or ceiling effects (Table 4).

**TABLE 4.** Floor and Ceiling Effects (n = 202) (%)

	Floor	Ceiling
PROMIS		
Physical function	0 (0)	0 (0)
Pain interference	0 (0)	0 (0)
SMFA		
Dysfunction index	0 (0)	5 (3)
Bother index	0 (0)	17 (8)

### Associated Factors

In the multivariable regression analysis, the strongest (independent) factors associated with worse (lower) PROMIS-PF scores were higher age, followed by lower extremity fracture, higher pain intensity, elevated level of depression, lower education level, and shorter follow-up period (Table 5). With respect to worse (higher) PROMIS-PI scores, associated factors were higher pain intensity, elevated level of depression, and lower extremity fracture. The full models accounted for 50% and 58% of the variance in PROMIS-PF and PROMIS-PI CAT scores, respectively.

**TABLE 5.** Multivariable Linear Regression Analysis

	PROMIS			
	Physical Function		Pain Interference	
	β Coefficient	*P*	β Coefficient	*P*
Mean age; y	−0.383	<0.001	0.012	0.789
Sex; male (y/n)	−0.073	0.189		
Education level; low (y/n)	−0.119	0.023		
Employment; paid job (y/n)	−0.007	0.908		
Fracture; upper extremity (y/n)	0.293	<0.001	−0.147	0.002
Treatment; operative (y/n)	−0.087	0.099		
Follow-up; d	0.118	0.022		
PROMIS depression	−0.265	<0.001	0.273	<0.001
Pain intensity	−0.266	<0.001	0.585	<0.001

Adjusted R^2^ for PROMIS-PF CAT: 0.499.

Adjusted R^2^ for PROMIS-PI CAT: 0.576.

## DISCUSSION

In this study, PROMIS-PF and PROMIS-PI CATs showed a (high and moderate) correlation with SMFA in patients treated for upper or lower extremity fractures, and, as such, provided a comparable measurement of overall physical functioning and discomfort. PROMIS CATs however require fewer items and much less time to complete, while maintaining strong reliability. This renders CATs much more efficient instruments to administer.

To our knowledge, no prior studies have analyzed the correlation of PROMIS (PF and PI) CATs with the SMFA (DI and BI) in a mixed group of orthopaedic trauma patients. Still, comparable findings were found in 3 (limited) samples of patients with distinct fracture types. In a cohort of 47 elderly patients with isolated upper extremity fractures, the correlation between PROMIS-PF CAT and SMFA-DI was high (r −0.81), and the correlation between PROMIS-PI and SMFA-BI was moderate (r −0.68).^12^ In another group of patients with isolated upper extremity fracture, PROMIS-PF (short form) and the SMFA-DI were highly correlated (r −0.80).^32^ Finally, in a group of 48 acetabular fracture patients, a strong correlation (r −0.84) was observed between PROMIS-PF CAT and SMFA-DI.^33^ Overall, these studies also found that specifically PROMIS-PF and SMFA-DI assess similar constructs.

The observed correlation between PROMIS-PI and SMFA-BI was not as high as between PROMIS-PF and SMFA-DI owing to important differences in content. PROMIS-PI is specifically designed to assess whether pain is limiting patients in performing certain activities. SMFA-BI, by contrast, evaluates whether any general (physical, mental, social) problem or symptom (pain, stiffness) is bothering them in certain functional areas.

The average number of items needed for test completion of PROMIS-PF and PROMIS-PI CATs (5 and 4, respectively) was considerably lower than the fixed 46 items required for the SMFA. Based on reliability analysis, PROMIS-PF and PROMIS-PI CATs were 8- and 4-times more efficient to administer than the SMFA. Correspondingly, the time needed for test completion was significantly shorter for PROMIS-PF and PROMIS-PI CATs when compared with the SMFA (roughly 1.5 and 1 minute vs. 6.5 minutes). This difference was somewhat smaller than found in earlier studies, which showed completion times for SMFA approaching up to 10 minutes longer than PROMIS.^10,12,33^

No floor or ceiling effects for PROMIS (PF and PI) CATs or the SMFA (DI and BI) were observed. This is similar to earlier reports, which found these effects to be (negligible or) absent for PROMIS-PF CAT in various cohorts of orthopaedic trauma patients.^10,12,33–37^ Still, some degree of ceiling effect has been found for the SMFA in the general orthopaedic trauma population.^13,15^ Ceiling effects were reported to be especially pronounced (up to 35%) in populations with certain injuries and in cohorts with prolonged durations of follow-up, limiting the ability of the SMFA to detect improvement over time.^10–12^

In terms of factors associated PROMIS CAT scores, the results showed an independent relationship between elevated levels of depression (among other factors) and worse physical function and pain interference scores. This is in accordance with growing evidence to support the critical importance of mental illness to poor outcomes in orthopaedic trauma patients.^38–43^ Notably, lower education level was also found to correlate with poor physical functioning, as has previously been reported.^38^

Limitations of this study include the fact that patients with weightbearing or motion restrictions were excluded to ensure that questions, such as those related to mobility, were (all) relevant. Also, results may not be applicable to multi-trauma patients. Furthermore, selection bias may have played a role as specific (elderly) patients, who lack digital literacy or proficiency to answer questions on a portable computer tablet, may have refused participation. Nonetheless, SMFA scores were comparable to other patients with musculoskeletal disorders.^9^ Lastly, the limited sample size may have impacted the (relative) efficiency calculation for the SMFA. The SMFA-DI and BI were however strongly unidimensional and distribution of item parameters was within normal limits, justifying the fitting of a graded response model and estimation of SEs.

Successful PROMs implementation requires efficient administration, as well as adequate patient engagement.^6,44^ Particularly in (busy) outpatient clinics, measurement data are ideally collected electronically, and results displayed on dashboards, rendering PROMs use more effective and less burdensome.^6^ Given that PROMIS CATs administer only relevant questions, take little time to complete, and generate easy to interpret results, these measures may be especially well suited for integration into the workflow of daily clinical practice. To be able to fully leverage this benefit, however, CATs are preferably incorporated into electronic health records, which continues to present a well-recognized challenge.^6,44^

In conclusion, PROMIS-PF and PROMIS-PI CATs showed a (high and moderate) correlation with SMFA in patients treated for isolated fractures, and hence measure comparable outcomes. PROMIS CATs are however more efficient to administer, significantly reducing the response burden for patients. As such, PROMIS-PF and PROMIS-PI CATs may present a compelling alternative to the traditional SMFA for evaluating the impact of fracture treatment. Among other factors, worse PROMIS (PF and PI) CAT scores were (independently) associated with elevated levels of depression, emphasizing the crucial role of psychosocial determinants of health in physical functioning of orthopaedic trauma patients.
